# Oppel – Kundt illusion manifestation among people with schizophrenia spectrum disorders

**DOI:** 10.1192/j.eurpsy.2022.1965

**Published:** 2022-09-01

**Authors:** A. Pečkutė, D. Leskauskas, A. Bulatov, E. Diržius

**Affiliations:** 1 Lithuanian University of Health Sciences, Department Of Psychiatry, Kaunas, Lithuania; 2 Lithuanian University of Health Sciences, Institute Of Biologal Systems And Genetic Research, Kaunas, Lithuania

**Keywords:** schizophrénia, Oppel – Kundt, visual perception

## Abstract

**Introduction:**

Various studies have reported differences in early visual processing, gain control and integration for patients with schizophrenia spectrum disorders (SSD). However, Oppel – Kundt (OK) illusion is not studied well enough among subjects affected by SSD. We decided to study the illusion to get more insights in visual perception for individuals with SSD.

**Objectives:**

To investigate the OK illusion manifestation among people with SSD.

**Methods:**

In the prospective study were included 15 patients, who were diagnosed with SSD and 15 matched comparison group (CG) without any mental, neurological diseases or visual impairment. OK figures used in the experiments consisted of three white spots presented horizontally against the black background. We used 3 different types of distractor stimuli – either straight one or two-sided line or circle. Using computerized equipment in OK figures the subjects were asked to adjust the unfilled part of the stimulus to be equal in length to the filled (referential) one. ANOVA, T-test and post hoc Bonferroni correction were used for statistical analysis.

**Results:**

People with SSD tended to make bigger mistakes when evaluating OK figures with the statistical difference which was the most eminent for the subgroup of individuals affected by paranoid schizophrenia. The manifestation of the illusion in the SSD group was contrariwise (respectively to zero point) to the CG group.

**Conclusions:**

OK illusion tended to manifest stronger for patients with SSD, this tendency was mostly expressed for the patients diagnosed with paranoid schizophrenia. The manifestation of OK illusion among persons with SSD was diametrically opposite to that seen for the healthy individuals.

**Disclosure:**

No significant relationships.

